# Characteristics of the human endometrial regeneration cells as a potential source for future stem cell-based therapies: A lab resources study

**DOI:** 10.18502/ijrm.v13i11.7961

**Published:** 2020-11-22

**Authors:** Fatemeh Akyash, Mahdieh Javidpou, Ehsan Farashahi Yazd, Jalal Golzadeh, Fatemeh Hajizadeh-Tafti, Reza Aflatoonian, Behrouz Aflatoonian

**Affiliations:** ^1^Stem Cell Biology Research Center, Yazd Reproductive Sciences Institute, Shahid Sadoughi University of Medical Sciences, Yazd, Iran.; ^2^Department of Reproductive Biology, School of Medicine, Shahid Sadoughi University of Medical Sciences, Yazd, Iran.; ^3^Department of Endocrinology and Female Infertility, Reproductive Biomedicine Research Center, Royan Institute for Reproductive Biomedicine, ACECR, Tehran, Iran.; ^4^Department of Advanced Medical Sciences and Technologies, School of Para Medicine, Shahid Sadoughi University of Medical Sciences, Yazd, Iran.

**Keywords:** Cell-based therapy, Endometrium, Mesenchymal stem/stromal cells, Regenerative medicine, Stem cells, Uterus.

## Abstract

**Background:**

Human endometrium with consecutive regeneration capability undergoes monthly hormonal changes for probable implantation, which confirms the presence of the cells in the basalis layer known as stem cell.

**Objective:**

Previously, we reported the isolation and culture of the mesenchymal-like cells from human endometrium. In this study, we evaluated the biological and stemness characteristics of these cells.

**Materials and Methods:**

The characterization of Yazd human endometrial-derived mesenchymal stem/stromal cells (YhEnMSCs) was assessed using immunofluorescence (IF) staining for CD105, VIMENTIN, and FIBRONECTIN as markers and RT-PCR for *CD166, CD10, CD105, VIMENTIN, FIBRONECTIN, MHCI*, *CD14*, and *MHCII* genes. Flow cytometry (FACS) was performed for CD44, CD73, CD90, and CD105 markers. Moreover, the differentiation capacity of the YhEnMSCs to the osteoblast and adipocytes was confirmed by Alizarin Red and Oil Red staining.

**Results:**

YhEnMSCs expressed CD105, VIMENTIN, FIBRONECTIN, CD44, CD73, and CD90 markers and *CD166, CD10, CD105, VIMENTIN, FIBRONECTIN, *and* MHCI,* but, did not express *CD14*, *MHCII*.

**Conclusion:**

Our data confirm previous reports by other groups indicating the application of endometrial cells as an available source of MSCs with self-renewal and differentiation capacity. Accordingly, YhEnMSCs can be used as a suitable source for cell-based therapies.

## 1. Introduction

Human endometrium is a unique tissue that undergoes a monthly cycle of regeneration and regression with each menstrual cycle of women. It regenerates the functionalis layer which is essential for a successful implantation; this procedure depends on the presence of the stem cell population that resides in the basalis layer of the endometrium (1, 2). Recently, high expression of *OCT-4, SOX-2*, *NANOG*, and *REX-1 *as stemness genes has been reported in the proliferative phase comprised of secretory and menstrual phases (3). The first evidence demonstrating the existence of the endometrial stem cells (EnSCs) was reported in 2004 (4). Following that, reports confirmed the mesenchymal stem cell (MSCs) properties of the endometrial-derived mesenchymal stem/stromal cells (EnMSCs) including self-renewal, proliferation, clonogenicity, and differentiation into different mesodermal cell lineages such as chondrocytes, osteocytes, adipocytes, and smooth muscle cells (5-9). Also, for the first time, Fayazi and colleagues demonstrated the differentiation ability of EnMSCs into neural and oligodendrocyte lineages (10). They used retinoic acid (RA) supplemented with epithelial growth factor (EGF) and basic fibroblast growth factor (bFGF) for the differentiation procedure and postulated that this potential depends on the essential role of RA in combination with growth factors (10). Similarly, other studies, besides clonality and multilineage differentiation potential, showed another MSC characteristics of endometrial-derived stromal cells such as the expression of CD146, CD105, CD90, CD73 as specific MSCs markers and the lack of hematopoietic markers expression including CD34 and CD14 (10, 11). Recently, reports confirmed expression of specific MSCs markers including CD73, CD105, CD29, CD44, differentiation potential into mesodermal cell lineages and also, cytokines secretion into isolated MSCs from menstrual blood (9). Therefore, owing to this potential, EnMSCs could be used as an appropriate source in the regenerative medicine and subsequent cell therapy for treating uterine-factor infertile patient in the future (1, 2, 7).

In this study, isolated and cultured spindle-shape human EnMSCs confirmed MSCs' properties by expression of specific markers including CD105, CD44, CD73, CD90, VIMENTIN, and FIBRONECTIN using immunofluorescence (IF) and flow cytometry (FACS) techniques. Moreover, reverse transcription-polymerase chain reaction (RT-PCR) was performed for *CD166, CD10, CD105, VIMENTIN, FIBRONECTIN, MHCI*, *CD14*, *MHCII* genes. Thereafter, osteogenic and adipogenic differentiation capability of the multipotent-derived EnMSCs were examined using Alizarin Red and Oil Red O staining, respectively.

## 2. Materials and Methods

The chemicals used in this lab resources study were purchased from Sigma Aldrich (Poole, UK), while the culture media and supplements were purchased from Invitrogen, Gibco (UK), unless otherwise stated.

### Samples collection and endometrial tissue preparation 

The collection of human endometrial tissues was performed at the Stem Cell Biology Research Center, Yazd Reproductive Sciences Institute, following a hysterectomy procedure of women aged 30-42 yr (n = 4) with polycystic ovarian syndrome (PCOs). At the beginning of the isolation of human endometrial cells for culture preparation, first, human endometrial tissues were washed in the Dulbecco's phosphate-buffered saline (PBS; Biowest) and dissected from myometrium layer. Next, tissue fragments were dissociated into single-cell suspension with mechanical and enzymatic digestion. Human EnMSCs and isolated cells were then cultured in the flasks (Falcon, USA) containing Dulbecco's modified Eagle's medium (DMEM) supplemented with 1 µl/ml penicillin/streptomycin and 10% fetal bovine serum (FBS; all from Gibco, Grand Island, NY) and finally incubated in a humidified atmosphere at 37°C and 5% CO${}_{2}$ as described previously (12).

### Immuno fluorescence localization for MSCs markers

IF staining was performed for characterization. For the IF technique, cells were washed thrice with PBS, then fixed with 4% paraformaldehyde (Sigma-Aldrich, St. Louis, MO, USA) solution for 15 min at room temperature. Next, the cells were washed twice for 5 min in PBS and treated with 0.1% Triton X-100 (Sigma, USA) for 5 min. The cells were then incubated for 30 min with PBS supplemented with 10% FBS as blocking solution for the inhibition of the nonspecific binding 24 hr before incubation with primary antibodies (CD105, VIMENTIN, and FIBRONECTIN) at 4°C. Incubation with secondary antibodies was performed for 1 hr at 37°C. Finally, inverted fluorescence microscopy (Olympus IX-71, Tokyo, Japan) was used for evaluating samples with appropriate excitation optics (13). It should be noted that negative controls were only incubated with secondary antibodies. Table I presents the information about the antibodies.

### RNA extraction, complementary DNA (cDNA) synthesis, and reverse-transcription PCR

Total RNA of the human EnMSCs was extracted by TRI Reagent (Sigma-Aldrich, USA) according to the manufacturer's instructions. The extracted RNA was then treated with DNase I (Invitrogen, USA), and complementary DNA (cDNA) synthesize was performed using the (Thermo scientific, USA) kit according to the manufacturer's protocol. Following that, the cDNA was subjected to RT-PCR in a 20-µl final reaction volume including 1 µl cDNA, 0.5 µl of each primer (20 mM), 6 µl dH${}_{2}$O, and 12 µl Taq DNA Polymerase Master Mix RED (Ampliqon, USA). The β*${}_{2}$-microglobulin* (β*${}_{2}$M)* gene was used as an internal control and target genes *CD166, CD10, CD105, VIMENTIN, FIBRONECTIN, MHCI*, *CD14*, *MHCII* were evaluated. The amplification plan was as follows: initial denaturation step at 94°C for 5 min, followed by another denaturation step at 94°C for 30 sec, annealing step at 58°C (*CD166, CD10, CD105, VIMENTIN, FIBRONECTIN, MHCI*, *CD14*, and* MHCII*), 60°C (*CD105 *and β*${}_{2}$M*) for 30 sec, an extension for 30 sec at 72°C for 40 cycles, and a final extension performed in two steps at 72°C for 5 min and 4°C for 1 min. PCR products were detected on 2% agarose gels electrophoresis. Table II presents the summary information of the primers.

### Characterization of Yazd human endometrial-derived mesenchymal stem/stromal cells (YhEnMSCs) using FACS

For the characterization of cultured cells using FACS, YhEnMSCs were washed with PBS containing 0.5% FBS after dissociation using 0.25% trypsin EDTA enzyme. Primary antibodies including CD44 (Monoclonal Antibody; FITC, eBioscience), CD73 (Monoclonal Antibody; PerCP, BD Bioscience), CD90 (Monoclonal Antibody; FITC, eBioscience), and CD105 (Monoclonal Antibody; PerCP, BD Bioscience) were used for 30 min and stored at 4°C. Next, the EnMSCs were incubated by appropriate fluorescent conjugated secondary antibodies in dark for 60 min at 4°C. While samples were analyzed on BD FACSCalibure (BD biosciences, San Jose, CA, USA), data analysis was performed using the FlowJo 7.6 software.

### Osteogenic and adipogenic differentiation of YhEnMSCs

To prove the stemness of the YhEnMSCs, osteogenic and adipogenic differentiation were induced in the EnMSCs. Osteogenic differentiation was performed using 3 × 10${}^{3}$ cells/cm${}^{2}$ which was cultured in DMEM + 10% FBS. At approximately 70% confluency, the osteogenic medium containing DMEM was supplemented with 50 mg/mL ascorbic acid 2-phosphate, 10 nM dexamethasone, and 10 mM b-glycerophosphate was replaced with DMEM + 10% FBS. The osteogenic medium was changed every three days for 21 days. After washing of the cells twice using PBS and fixation by paraformaldehyde 4% (Sigma) for 30 min, matrix mineralization was stained by 40 mM Alizarin Red for 15 min. For adipogenesis, EnMSCs were cultured with differentiation medium containing DMEM supplemented with 50 mg/mL indomethacin and 100 nM dexamethasone for 21 days which was changed every three days. For the detection of lipid droplets, cells were stained by Oil Red O. These differentiation experiments were performed in triplicate (13).

### Karyotype analysis of YhEnMSCs 

The chromosomal content of the YhEnMSCs was determined by standard G-banding procedure which has been described previously (14). G-bandings were analyzed through light microscopy (Ziess, Germany, Axiophot) using applied spectral imaging software.

**Table 1:** List of antibodies used for IF


**Primary antibody**			**Secondary antibody**		
**Name**	**Dilution**	**Catalog number**	**Type**	**Dilution**	**Catalog number**
**CD105/EGLN1**	1:100	Abcam ab11414	Anti-mouse IgG (FITC)	1:100	Abcam ab6785
**VIMENTIN**	1:100	Millipore AB573	Anti-Chicken IgY (TR)	1:100	Abcam ab6751
**FIBRONECTIN**	1:200	Abcam ab6328	Anti-mouse IgG (FITC)	1:100	Abcam ab6785
FITC: Fluorescein isothiocyanate; TRITC; TR: Tetramethylrhodamine					

**Table 2:** List of primers used for RT-PCR


**Gene**	**Forward primer (5' →3')**	**Reverse primer (5' →3')**	**Annealing temp (°C)**	**Prod size (bp)**
*CD105*	CTTGGCCTACAATTCCAGCC	CTTGAGGTGTGTCTGGGAGC	60	542
*CD166*	TCCTGCCGTCTGCTCTTCT	TTCTGAGGTACGTCAAGTCGG	58	128
*CD10*	GGCACCAGAAGAACAGTAGG	ATCTCAGCATCAGTCAAAGC	58	269
*FIBRONECTIN*	AGGAAGCCGAGGTTTTAACTG	AGGACGCTCATAAGTGTCACC	58	106
*VIMENTIN*	TCTATCTTGCGCTCCTGAAAAACT	AAACTTTCCCTCCCTGAACCTGAG	58	270
*CD14*	CACACTCGCCTGCCTTTTCC	GATTCCCGTCCAGTGTCAGG	58	450
*MHCI*	CCTACGACGGCAAGGATTAC	TGCCAGGTCAGTGTGATCTC	58	304
*MHCII*	GCCGAGTTCTATCTGAATCCTG	TTGCGCAATCCCTTGATGATG	58	629
*$\beta_{2}$M*	AGATGAGTATGCCTGCCGTG	TGCGGCATCTTCAAACCTC	60	106

### Ethical consideration

The YhEnMSCs were isolated and cultured after obtaining fully informed written consent from the participants prior to the study, in compliance with the guidelines of the Shahid Sadoughi University of Medical Sciences Ethical Committee (code: IR.SSU.REC.1396.186).

## 3. Results 

### Isolation and culture of YhEnMSCs

Monitoring of the cell adherence and growth were performed 24 hr after the culture by inverted microscopy (Olympus CKX-41, Tokyo, Japan); some of the adherent cells were detected on the bottom of the flask. During several passages, homogenous EnMSCs fibroblast-like cells were observed (12). Moreover, colonies were formed during the passaging of the cells in different passage numbers (12). In addition, during the colony formation, various morphological cell types from spindle-shaped to triangular patterns could be detected.

### Characterization of YhEnMSCs using IF 

**Figure 1:**
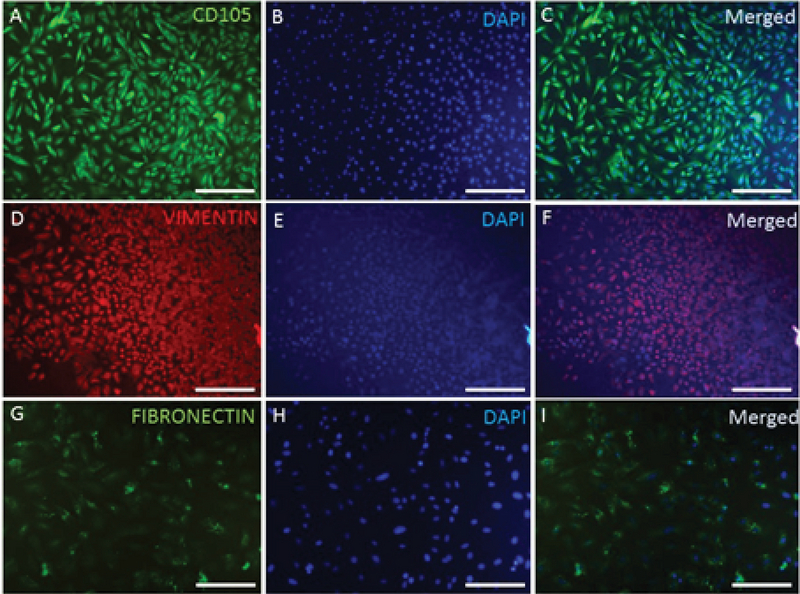
Characterization of YhEnMSCs using IF staining. CD105 (A), DAPI (B), and CD105 and DAPI merged (C). VIMENTIN (D), DAPI (E), and VIMENTIN and DAPI merged (F). FIBRONECTIN (G), DAPI (H), and FIBRONECTIN and DAPI merged (I). Scale bars: A-F: 200 µm, G-I: 100 µm.

The expression of the specific EnMSC markers was examined by IF technique with CD105, VIMENTIN, and FIBRONECTIN antibodies. Human EnMSCs were CD105-positive (Figures 1A, B, and C), and also, the expression of the VIMENTIN (Figure 1D, E, and F) and FIBRONECTIN (Figure 1G, H, and I) markers was detected in the human EnMSCs.

### Gene expression profile of YhEnMSCs 

**Figure 2:**
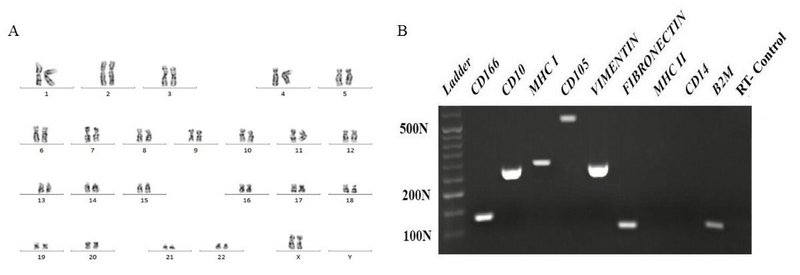
A normal, diploid 46, XX karyotype for the YhEnMSCs (A) was shown using G-binding. Further characterization of YhEnMSCs using RT-CR. Yazd hEnMSCs revealed their gene expression profile for specific genes such as* CD166, CD10, CD105, VIMENTIN, FIBRONECTIN, *and* MHCI. *Cells did not express* CD14 *and* MHCII *(B).

Yazd hEnMSCs with normal karyotype (46 XX; Figure 2A) expressed the MSCs genes including *CD166, CD10, CD105, VIMENTIN, FIBRONECTIN, MHCI *(Figure 2B). However,* CD14 *and* MHCII *were not expressed (Figure 2B).

### Characterization of YhEnMSCs using FACS

**Figure 3:**
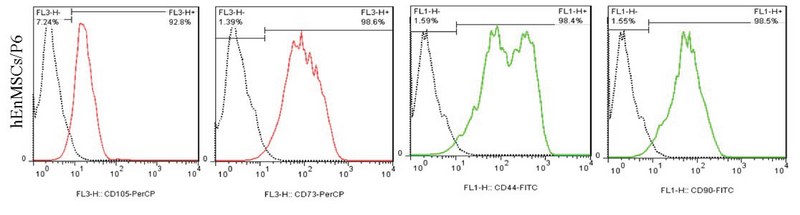
Characterization of YhEnMSCs using FACS assessment indicated that YhEnMSCs were positive for CD44, CD73, CD90, and CD105.

Yazd EnMSCs expressed CD105, CD90, CD73, and CD44 as specific MSCs markers using FACS (Figure 3).

### *In vitro* differentiation of YhEnMSCs

**Figure 4:**
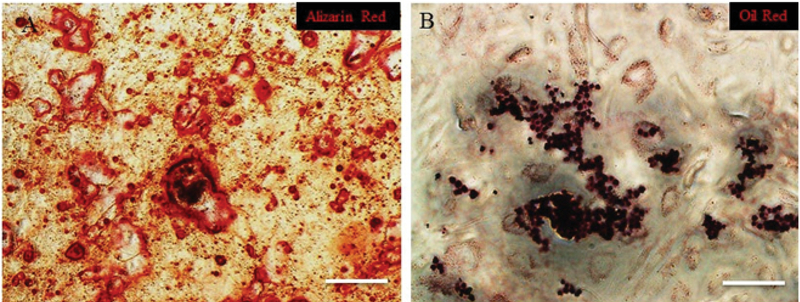
Differentiation capacity of YhEnMSCs. Osteogenic (A) and adipogenic (B) differentiation of YhEnMSCs was confirmed by Alizarin Red and Oil Red staining. Scale bars: 100 µm.

Matrix mineralization and accumulation of the lipid droplets after differentiation into osteogenic (Figure 4A) and adipogenic (Figure 4B) lineages were shown respectively.

## 4. Discussion

Our data confirm the previously reported findings for the identification of MSCs from human endometrium using specific genes expression profiles and markers. Moreover, the cells show a differentiation capacity to osteoblasts and adipocytes.

Similar findings reported positive expression of the MSCs specific markers using FACS analysis for stromal cells colonies-derived endometrium including CD29, CD44, CD73, CD105, CD140B (6). Another study showed positive expression of CD90 and CD105 markers and reported that CD146 was significantly positive in cultured cells which are known as mesenchymal stromal stem cells marker (10).

Previously (4, 9), the clonogenicity ability of the stromal cells derived from endometrium was confirmed in a similar way as that of the YhEnMSCs behavior in this study (12). Moreover, similar to other studies (5-9), we demonstrated the differentiation potential of YhEnMSCs into adipogenic and osteogenic lineages.

Interestingly, studies showed immunomodulatory effects of MSCs due to lack of MHC class II expression which inhibited T cell proliferation (15). On the other hand, MSCs present positive expression of major histocompatibility complex (MHC) class I during differentiation into adipogenic, osteogenic, and chondrogenic lineages that protect them from Natural killer (NK) cells mechanisms (16). Therefore, the immunomodulatory feature of MSCs makes them highly attractive for clinical applications. Our results showed positive and negative expression of MHC class I and II, respectively.

For clinical application of hEnMSCs, Zhang and colleagues reported positive effect of conditioned medium from human endometrial mesenchymal stem cells derived from menstrual blood (MenSCs) on angiogenesis (17). Also, recently, it was shown that EnMSCs improved cardiac function in infarcted rat myocardium (18). Interestingly, Wang and colleagues explored that MenSCs can be regenerated and could improve ovarian function in mice with a premature ovarian failure (19). It should also be noted that the differentiation potential of EnMSCs can be used for repairing various types of tissue injury.

In sum, the isolated YhEnMSCs (12) were characterized with specific MSCs markers CD105, CD44, CD73, CD90, VIMENTIN, and FIBRONECTIN using IF and FACS. In addition, the gene expression profile of the cells for *CD166, CD10, CD105, VIMENTIN, FIBRONECTIN, MHCI* genes was evaluated using RT-PCR. YhEnMSCs did not express *CD14* and *MHCII*. Osteogenic and adipogenic differentiation potential of the YhEnMSCs was confirmed using Alizarin Red and Oil Red staining.

## 5. Conclusion

In conclusion, the endometrial tissue as a part of uterus with self-renewal and differentiation capacity that undergoes a cyclical regeneration every month in normal women's life span is a source of MSCs. YhEnMSCs can proliferate and differentiate into different cell types. Hence, according to the therapeutic application of these cells, YhEnMSCs could be used for future novel therapeutic methods in regenerative medicine for the treatment of uterine-factor infertile patients which can lead to recurrent pregnancy loss (RPL) and finally resolve surrogacy problems.

##  Conflict of Interest 

The authors declare no conflicts of interest.

## References

[1] Gargett CE. Uterine stem cells: what is the evidence? *Hum Reprod Update* 2007; 13: 87–101. 10.1093/humupd/dml045 16960017

[2] Gargett CE, Chan RW, Schwab KE. Hormone and growth factor signaling in endometrial renewal: role of stem/progenitor cells. *Mol Cell Endocrinol* 2008; 288: 22–29. 10.1016/j.mce.2008.02.026 18403104

[3] Shariati F, Favaedi R, Ramazanali F, Ghoraeian P, Afsharian P, Aflatoonian B, et al. Increased expression of stemness genes *REX-1*, *OCT-4*, *NANOG*, and *SOX-2* in women with ovarian endometriosis versus normal endometrium: A case-control study. *Int J Reprod Biomed* 2019; 16: 783–790. 10.18502/ijrm.v16i12.3684 PMC6600279 31417979

[4] Chan RW, Schwab KE, Gargett CE. Clonogenicity of human endometrial epithelial and stromal cells. *Biol Reprod* 2004; 70: 1738–1750. 10.1095/biolreprod.103.024109 14766732

[5] Schwab KE, Chan RW, Gargett CE. Putative stem cell activity of human endometrial epithelial and stromal cells during the menstrual cycle. *Fertil Steril* 2005; 84 (Suppl.): 1124–1130. 10.1016/j.fertnstert.2005.02.056 16210003

[6] Gargett CE, Schwab KE, Zillwood RM, Nguyen HP, Wu D. Isolation and culture of epithelial progenitors and mesenchymal stem cells from human endometrium. *Biol Reprod* 2009; 80: 1136–1145. 10.1095/biolreprod.108.075226 PMC2849811 19228591

[7] Cervelló I, Gil-Sanchis C, Mas A, Delgado-Rosas F, Martínez-Conejero JA, Galán A, et al. Human endometrial side population cells exhibit genotypic, phenotypic and functional features of somatic stem cells. *PLoS One* 2010; 5: e10964. 1–15. 10.1371/journal.pone.0010964 PMC2891991 20585575

[8] Shoae-Hassani A, Sharif Sh, Seifalian AM, Mortazavi-Tabatabaei SA, Rezaie S, Verdi J. Endometrial stem cell differentiation into smooth muscle cell: a novel approach for bladder tissue engineering in women. *BJU Int* 2013; 112: 854–863. 10.1111/bju.12195 24028767

[9] Du X, Yuan Q, Qu Y, Zhou Y, Bei J. Endometrial mesenchymal stem cells isolated from menstrual blood by adherence. *Stem Cells Int* 2016; 2016: 3573846. 10.1155/2016/3573846 PMC4670906 26681948

[10] Fayazi M, Salehnia M, Ziaei S. Differentiation of human CD146-positive endometrial stem cells to adipogenic, osteogenic, neural progenitor, and glial-like cells. *In Vitro Cell Dev Biol Animal *2015; 51: 408–414. 10.1007/s11626-014-9842-2 25515247

[11] Schüring AN, Schulte N, Kelsch R, Röpke A, Kiesel L, Götte M. Characterization of endometrial mesenchymal stem-like cells obtained by endometrial biopsy during routine diagnostics. *Fertil Steril* 2011; 95: 423–426. 10.1016/j.fertnstert.2010.08.035 20864098

[12] Akyash F, Javidpou M, Aflatoonian A, Aflatoonian B. Isolation and culture of human endometrial derived cells as an in vitro model for future implantation studies. *J Shahid Sadoughi Uni Med Sci* 2019; 27: 1584–1590.

[13] Sadeghian-Nodoushan F, Aflatoonian R, Borzouie Z, Akyash F, Fesahat F, Soleimani M, et al. Pluripotency and differentiation of cells from human testicular sperm extraction: An investigation of cell stemness. *Mol Reprod Dev* 2016; 83: 312–323. 10.1002/mrd.22620 27077675

[14] Akyash F, Tahajjodi SS, Farashahi Yazd E, Hajizadeh-Tafti F, Sadeghian-Nodoushan F, Golzadeh J, et al. Derivation of new human embryonic stem cell lines (Yazd1-3) and their vitrification using Cryotech and Cryowin tools: A lab resources report. *Int J Reprod BioMed *2019; 17: 891– 906. 10.18502/ijrm.v17i12.5808 PMC6943792 31970311

[15] Krampera M, Glennie S, Dyson J, Scott D, Laylor R, Simpson E, et al. Bone marrow mesenchymal stem cells inhibit the response of naive and memory antigen-specific T cells to their cognate peptide. *Blood* 2003; 101: 3722–3729. 10.1182/blood-2002-07-2104 12506037

[16] Machado Cde V, Telles PD, Nascimento IL. Immunological characteristics of mesenchymal stem cells. *Rev Bras Hematol Hemoter* 2013; 35: 62–67. 10.5581/1516-8484.20130017 PMC3621638 23580887

[17] Zhang Y, Lin X, Dai Y, Hu X, Zhu H, Jiang Y, et al. Endometrial stem cells repair injured endometrium and induce angiogenesis via AKT and ERK pathways. *Reproduction* 2016; 152: 389–402. 10.1530/REP-16-0286 27486270

[18] Wang K, Jiang Zh, Webster KA, Chen J, Hu H, Zhou Y, et al. Enhanced cardioprotection by human endometrium MSCs driven by exosomal microRNA-21. *Stem Cells Transl Med* 2017; 6: 209–222. 10.5966/sctm.2015-0386 PMC5442741 28170197

[19] Wang Zh, Wang Y, Yang T, Li J, Yang X. Study of the reparative effects of menstrual-derived stem cells on premature ovarian failure in mice. *Stem Cell Res Ther* 2017; 8: 11–24. 10.1186/s13287-016-0458-1 PMC5259841 28114977

